# Comparative effects of different sport psychology interventions on anxiety in competitive athletes: a Bayesian network meta-analysis

**DOI:** 10.3389/fpsyg.2026.1842687

**Published:** 2026-08-12

**Authors:** Zhongjun Chen, Ling Lian, Songwei Zhong

**Affiliations:** Faculty of Sports and Health Sciences, Guangxi Science & Technology Normal University, Laibin, China

**Keywords:** anxiety, Bayesian network meta-analysis, cognitive-behavioral therapy, competitive athletes, sport psychology interventions

## Abstract

**Background:**

Anxiety is a prominent psychological factor that can impair both training adaptation and competitive performance in athletes. Although sport psychology interventions are generally considered beneficial for anxiety reduction, the comparative effectiveness of different intervention approaches remains unclear.

**Objective:**

To systematically evaluate the effects of sport psychology interventions on anxiety in competitive athletes and to compare their relative efficacy using Bayesian network meta-analysis.

**Methods:**

Randomized controlled trials (RCTs) examining sport psychology interventions for anxiety reduction in competitive athletes were systematically identified. Pairwise meta-analysis was conducted to estimate the overall effect, with sensitivity analysis, subgroup analysis, and meta-regression performed to explore the robustness of findings and potential sources of heterogeneity. Bayesian network meta-analysis was then used to compare and rank the relative effects of different interventions.

**Results:**

Twenty-four studies were included. Pairwise meta-analysis showed that sport psychology interventions significantly reduced anxiety in competitive athletes compared with control conditions (SMD = −0.74, 95% CI: −1.05 to −0.43; *p* < 0.0001), and sensitivity analyses supported the robustness of this finding. Subgroup analyses suggested that both individual and group-based interventions were effective, with generally consistent effects across competitive levels. Intervention timing emerged as the only significant subgroup factor, with interventions delivered during the regular training period showing the largest effect (SMD = −0.94, 95% CI: −1.43 to −0.45), while immediate pre-competition interventions also showed a stable benefit (SMD = −0.46, 95% CI: −0.58 to −0.33). Meta-regression did not identify significant linear associations between the examined covariates and effect sizes. In the Bayesian network meta-analysis, arousal regulation interventions (AR), cognitive-behavioral therapy (CBT), and mindfulness/acceptance/meditation-based interventions (MAM) showed significant advantages over passive control (PC), and CBT had the highest probability of being ranked first (86.14%).

**Conclusion:**

Sport psychology interventions appear effective overall for reducing anxiety in competitive athletes. Different intervention approaches may vary in relative efficacy, with CBT, AR, and MAM emerging as the most promising options and CBT showing the greatest likelihood of relative advantage within the current evidence base. Intervention timing may also be an important factor influencing treatment effects.

**Systematic review registration:**

https://www.crd.york.ac.uk/PROSPERO/view/CRD420261347470, identifier CRD420261347470.

## 1 Introduction

In competitive sport, anxiety is a salient psychological factor that can influence both athletes’ adaptation to training and their performance in competition. Athletes across competitive levels–from elite performers to collegiate and youth athletes still undergoing athletic and psychosocial development–may be chronically exposed to multiple stressors, including performance expectations, competitive pressure, pre-competition uncertainty, and external evaluation, which may increase their vulnerability to anxiety-related symptoms (Reardon et al., 2019). Previous research has shown that the pooled prevalence of anxiety- or depression-related symptoms among elite athletes may be as high as 34% (Gouttebarge et al., 2019), underscoring the importance of mental health concerns in competitive sport populations. More importantly, anxiety is not merely a negative emotional state. When it becomes excessive or is poorly regulated, it may disrupt attentional control, impair decision-making and motor execution, and intensify in the period preceding competition, thereby increasing the likelihood of performance decrements under pressure (Hufton et al., 2026; Hanton et al., 2004). Accordingly, how to effectively identify and alleviate anxiety in competitive athletes, so as to reduce its detrimental effects on training adaptation and competitive performance, has become an important concern for both sport psychology research and applied practice.

To address this issue, researchers have developed a range of sport psychology interventions, including mindfulness training, relaxation training, imagery training, self-talk, cognitive-behavioral interventions, and mental skills training (Xie et al., 2025; Lange-Smith et al., 2024). These approaches should not be viewed as homogeneous strategies; rather, they differ in their theoretical underpinnings, mechanisms of action, and modes of delivery, and may therefore vary in their efficacy for reducing anxiety. For example, cognitive-behavioral interventions primarily aim to modify maladaptive cognitive appraisals, mindfulness-based interventions emphasize attentional regulation and acceptance, whereas relaxation-based interventions primarily reduce physiological arousal. These theoretical differences suggest that intervention effectiveness may vary across approaches and therefore warrant direct comparison. Existing studies generally support the beneficial effects of sport psychology interventions on athletes’ anxiety and related psychological outcomes (Wang et al., 2025; Li et al., 2025). However, such conclusions have largely been derived from individual trials or from comparisons between a single intervention and a control condition.

This limitation is also reflected in the current synthesis literature. Existing systematic reviews and meta-analyses have primarily examined either the overall effectiveness of psychological interventions or individual intervention modalities, such as mindfulness-based interventions and psychological skills training (Ong and Chua, 2021; Wang et al., 2023; Birrer and Morgan, 2010). Although these reviews consistently suggest that psychological interventions are beneficial for reducing competitive anxiety, their findings are difficult to translate into intervention selection because they provide only limited evidence regarding the comparative effectiveness of different intervention approaches. Most previous evidence syntheses relied on conventional pairwise meta-analysis or focused on a single intervention category, making it difficult to simultaneously compare multiple competing interventions or estimate their relative effectiveness when direct comparisons are unavailable. Moreover, substantial heterogeneity exists across studies with respect to participant characteristics, competitive level, intervention duration, delivery format, and anxiety assessment methods (Nien et al., 2023; Li et al., 2023). Such variability complicates the interpretation of pooled estimates and reduces confidence in identifying genuine differences in intervention effectiveness. Furthermore, relatively few evidence syntheses have systematically examined whether intervention effectiveness varies according to competitive level, intervention timing, or delivery format, despite the fact that competitive anxiety is inherently context-dependent and phase-specific. Consequently, despite the growing body of evidence supporting sport psychology interventions, current evidence remains insufficient to determine the comparative effectiveness of different intervention approaches or to provide clear evidence for intervention selection in specific competitive sport settings.

Given the coexistence of multiple candidate interventions and the scarcity of direct head-to-head comparisons, a Bayesian network meta-analysis is particularly well suited to this field because it can integrate both direct and indirect evidence within a single analytical framework, estimate the relative efficacy of competing interventions, and generate probabilistic rankings. Therefore, this study conducted a Bayesian network meta-analysis to compare and rank the effects of different sport psychology interventions on anxiety in competitive athletes. Specifically, we aimed to estimate the relative effectiveness of competing intervention strategies and provide evidence to support intervention selection in competitive sport settings.

## 2 Methods

### 2.1 Study design and registration

This meta-analysis was conducted in accordance with the Preferred Reporting Items for Systematic Reviews and Meta-Analyses (PRISMA 2020) guidelines for systematic reviews and network meta-analyses and was prospectively registered in the PROSPERO database (CRD420261347470) (Page et al., 2021). No deviations from the registered PROSPERO protocol occurred during the conduct of this review.

### 2.2 Literature search strategy

A systematic literature search was performed in PubMed, Web of Science, Scopus, Embase, PsycINFO, China National Knowledge Infrastructure (CNKI), and VIP Database from database inception to March 15, 2026. In addition, the reference lists of all included studies and relevant review articles were manually screened to identify potentially eligible studies that may have been missed in the electronic search. Gray literature (e.g., conference abstracts, and preprints) was not searched because these sources often lack peer review and sufficient methodological information for reliable effect size estimation. The search strategy combined controlled vocabulary terms and free-text keywords and was developed around the following core concepts: competitive athletes, sport psychology interventions, anxiety, and randomized controlled trials. Search terms were adapted as appropriate for the indexing characteristics and search requirements of each database. The full search strategies for all databases are provided in the Supplementary Material. Study selection was conducted independently by two reviewers (CZJ and LL) according to the prespecified eligibility criteria. Any disagreements were resolved through discussion, and when necessary, consultation with a third reviewer (ZSW) (Supplementary Additional File 1).

### 2.3 Eligibility criteria

Eligibility criteria were established according to the PICOS framework.

Inclusion criteria

Studies were included if they met all of the following criteria:

a. Population (P): competitive athletes, defined as individuals who regularly participated in organized sports competitions at the youth, collegiate, amateur, or elite/professional level. Athletes were required to be actively engaged in structured training and formal competition during the study period, regardless of sex or sport type;
b. Intervention (I): Interventions were classified according to their primary theoretical framework and core therapeutic components: AR emphasized acceptance and defusion; CBT focused on cognitive restructuring; MAM integrated mindfulness with acceptance; psychological skills training (PST) targeted skill acquisition (e.g., goal-setting, self-talk, imagery); and multicomponent interventions (MCI) involved mental rehearsal or imagery. When an intervention combined techniques from multiple approaches without a clearly dominant orientation, it was classified as a multicomponent intervention. The detailed classification rationale for each study, including extracted components and assignment justification, is provided in Supplementary Additional File 3.
c. Comparator (C): usual training, no intervention, waitlist control, placebo/sham intervention, or another sport psychology intervention;
d. Outcomes (O): at least one anxiety-related outcome, such as competitive state anxiety, state anxiety, or trait anxiety, with sufficient data available to calculate effect sizes;
e. Study design (S): randomized controlled trials (RCTs).

Only studies published in English or Chinese were considered eligible.

Exclusion criteria

Studies were excluded if they met any of the following criteria:

a. The sample did not consist of competitive athletes;
b. The intervention was not primarily psychological in nature or could not be classified as a clearly defined sport psychology intervention;
c. Anxiety-related outcomes were not reported, or data were insufficient for effect size calculation;
d. The publication was a review, conference abstract, thesis/dissertation, case report, commentary, or study protocol;
e. The study was a duplicate publication, in which case the report with the most complete information was retained;

### 2.4 Data extraction

Data extraction was performed independently by two reviewers (CZJ and LL) according to a prespecified data extraction form, and the extracted information was cross-checked for accuracy. Any discrepancies were resolved through discussion, and if necessary, by consultation with a third reviewer (ZSW). The extracted data primarily included general study characteristics, study design and sample characteristics, participants’ age and sex distribution, intervention characteristics, and outcome measures.

### 2.5 Risk of bias assessment

The methodological quality of the included randomized controlled trials was independently assessed by two reviewers using the Cochrane Risk of Bias 2 tool (RoB 2). Any disagreements were resolved through discussion or, when necessary, adjudicated by a third reviewer. RoB 2 evaluates bias across five domains: bias arising from the randomization process, bias due to deviations from intended interventions, bias due to missing outcome data, bias in outcome measurement, and bias in the selection of the reported result. Each study was judged as having low risk of bias, some concerns, or high risk of bias (Sterne et al., 2019). The results of the RoB 2 assessment were used to characterize the methodological quality of the included studies and to inform the interpretation of the findings, rather than as a criterion for study exclusion or statistical weighting.

### 2.6 Statistical analysis

The standardized mean difference (SMD) and its corresponding standard error (SE) were calculated using Hedges’ g in order to standardize continuous outcomes measured on different scales. Ninety-five percent confidence intervals (CIs) or 95% credible intervals (CrIs) were used to quantify the uncertainty of effect estimates. All raw data were first processed in STATA 14 to calculate SMDs and SEs and were then organized into the format of relative effect estimates (diff) and their standard errors (std.err) before being imported into R software (version 4.5.1) for further analysis (Supplementary Additional File 2).

Because the gemtc package models continuous outcomes using mean differences (MDs) by default, but its contrast-based model is insensitive to the scale of the effect measure, the pre-calculated SMDs were entered into the model as MDs for the Bayesian network meta-analysis. All pairwise meta-analyses were conducted in R, and the Bayesian network meta-analysis was performed using relevant Bayesian modeling procedures in R. Two-sided *P*-values were reported only for the conventional pairwise meta-analysis, subgroup analyses, and meta-regression, whereas Bayesian network meta-analysis results were interpreted using posterior estimates and 95% credible intervals (CrIs).

For the conventional pairwise meta-analysis, a random-effects model was used to account for potential heterogeneity across studies in terms of sample characteristics, intervention content, and outcome measurement tools. Between-study heterogeneity was assessed using Cochran’s Q test and the I^2^ statistic, with I^2^ > 50% indicating moderate to substantial heterogeneity. To examine the robustness of the pooled results, leave-one-out sensitivity analyses were conducted. In addition, subgroup analyses were performed according to intervention delivery format, competitive level, and intervention timing to explore potential effect modifiers. Meta-regression analyses were also conducted to examine the linear associations between study-level covariates and effect sizes. Both subgroup analyses and meta-regression were considered exploratory analyses.

Because network meta-analysis relies on the assumption of transitivity, we carefully evaluated the clinical and methodological comparability of the included studies before conducting the analysis. Studies were eligible only if they involved competitive athletes, evaluated sport psychology interventions targeting anxiety, and reported comparable anxiety-related outcomes. Although intervention content and participant characteristics varied across studies, these differences were considered clinically acceptable because all interventions shared the common objective of reducing anxiety in competitive athletes. The plausibility of the transitivity assumption was further evaluated by comparing key clinical and methodological characteristics (e.g., competitive level, intervention duration, delivery format, and anxiety outcome measures) across treatment comparisons.

Building on the pairwise meta-analysis, a Bayesian network meta-analysis was performed to compare the effects of different sport psychology interventions on anxiety in competitive athletes. The SMD and its 95% CrI were used as the effect measure, with SMD < 0 indicating that the intervention favored a reduction in anxiety. Network meta-analysis allows the simultaneous integration of direct and indirect evidence, thereby enabling a comprehensive comparison of multiple competing interventions. Non-informative prior distributions were specified for all treatment effects and the between-study heterogeneity parameter. Model parameters were estimated using Markov chain Monte Carlo (MCMC) methods. Bayesian network meta-analysis was conducted using a consistency model with a random-effects specification. Model parameters were estimated using Markov chain Monte Carlo simulations with four parallel chains, 20,000 adaptation iterations, and 50,000 sampling iterations, with a thinning interval of 1. Model convergence was primarily assessed by trace plots, density plots, and the potential scale reduction factor (PSRF), with PSRF values close to 1.00 indicating satisfactory convergence. In addition, ranking probabilities and the surface under the cumulative ranking curve (SUCRA) were used to rank the relative effectiveness of the interventions, with higher SUCRA values indicating a greater likelihood of superiority in reducing anxiety. Comparison-adjusted funnel plots were used to assess potential publication bias and small-study effects.

## 3 Results

### 3.1 Study selection

**FIGURE 1:**
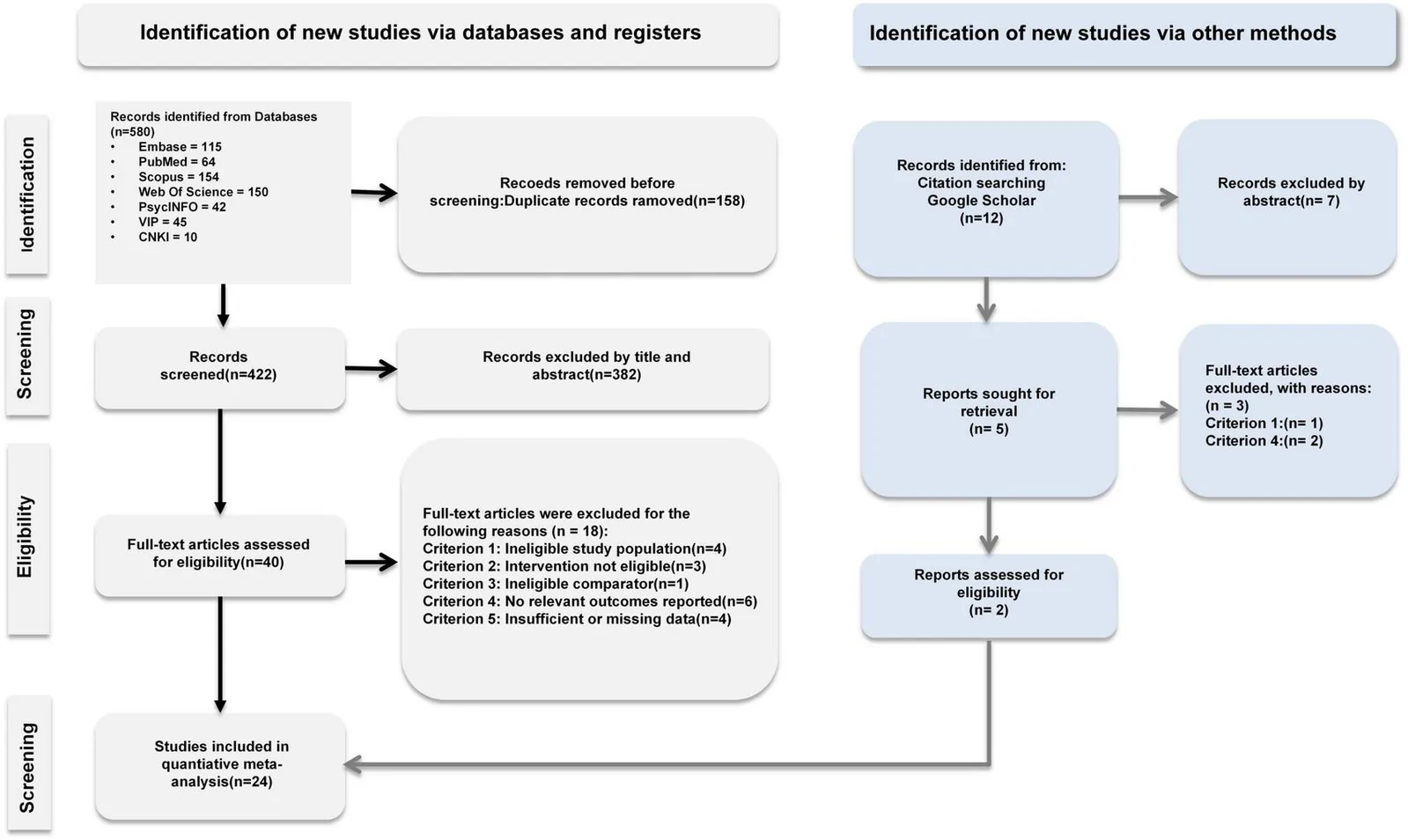
PRISMA flow diagram of study selection.

The literature search of seven electronic databases (Web of Science, Scopus, PubMed, PsycINFO, Embase, CNKI, and VIP Database) identified 580 records. After removal of 158 duplicate records, the remaining studies were screened against the predefined eligibility criteria. Of these, 382 records were excluded after title and abstract screening, and 18 studies were excluded following full-text review. In addition, 2 eligible studies were identified through Google Scholar searches and citation tracking. A total of 24 studies were ultimately included in this review and meta-analysis (Figure 1).

### 3.2 Study characteristics

A total of 24 studies published between 1995 and 2025 were included in the review and meta-analysis (Ali, 2015; Barlow and Banks, 2014; Barnicle and Burton, 2016; Bordo et al., 2025; Chen et al., 2024; Dehghani et al., 2018; Fortes et al., 2016; Gao et al., 2024; Georgakaki and Karakasidou, 2017; Davisson, 2008; Hatzigeorgiadis et al., 2009; Kanniyan, 2015; Liang et al., 2021; Paul and Garg, 2012; Sánchez-Sánchez et al., 2023; Scott-Hamilton et al., 2016; Terry et al., 1995; Tóth et al., 2023; Walter et al., 2019; Yu et al., 2024; Bu et al., 2020; Jiang, 2000; Liu, 2023; Liu et al., 2021). Overall, the number of publications increased in recent years, with a substantial proportion published after 2020 (Bordo et al., 2025; Chen et al., 2024; Gao et al., 2024; Liang et al., 2021; Sánchez-Sánchez et al., 2023; Tóth et al., 2023; Yu et al., 2024; Liu, 2023; Liu et al., 2021). The included studies were conducted across diverse countries and regions, including China, the United States, the United Kingdom, Italy, Iran, Brazil, Greece, India, Spain, Australia, Germany, Hungary, Saudi Arabia, and Algeria, with a relatively larger number originating from China (Chen et al., 2024; Gao et al., 2024; Liang et al., 2021; Yu et al., 2024; Bu et al., 2020; Liu, 2023; Liu et al., 2021). The total sample comprised 1306 competitive athletes, with participant ages ranging from approximately 11–40 years. Most studies focused on adolescent and young adult athletes (Barlow and Banks, 2014; Dehghani et al., 2018; Fortes et al., 2016; Davisson, 2008; Hatzigeorgiadis et al., 2009; Kanniyan, 2015; Liang et al., 2021; Paul and Garg, 2012; Sánchez-Sánchez et al., 2023; Scott-Hamilton et al., 2016; Terry et al., 1995; Tóth et al., 2023; Walter et al., 2019; Yu et al., 2024; Jiang, 2000; Liu, 2023; Liu et al., 2021). The included samples covered a broad range of competitive levels, from international/national elite athletes to professional, collegiate, club-level, youth, and interscholastic athletes, although collegiate and youth competitive athletes were more frequently represented. The included studies involved both individual and team sports. Individual sports slightly outnumbered team sports and included disciplines such as tennis, swimming, track and field, cycling, Latin dance, and sailing/windsurfing (Bordo et al., 2025; Chen et al., 2024; Fortes et al., 2016; Georgakaki and Karakasidou, 2017; Davisson, 2008; Hatzigeorgiadis et al., 2009; Kanniyan, 2015; Liang et al., 2021; Terry et al., 1995; Yu et al., 2024; Jiang, 2000; Liu et al., 2021), whereas team sports included football, netball, basketball, and ice hockey (Ali, 2015; Barlow and Banks, 2014; Barnicle and Burton, 2016; Dehghani et al., 2018; Kanniyan, 2015; Paul and Garg, 2012; Tóth et al., 2023; Liu, 2023). Several studies also enrolled mixed-sport samples (Gao et al., 2024; Sánchez-Sánchez et al., 2023; Walter et al., 2019; Bu et al., 2020).

According to their primary theoretical orientation and core techniques, the interventions were grouped into five categories: relaxation/arousal regulation (Ali, 2015; Liang et al., 2021; Paul and Garg, 2012; Terry et al., 1995; Jiang, 2000), psychological skills training (Barnicle and Burton, 2016; Fortes et al., 2016; Davisson, 2008; Hatzigeorgiadis et al., 2009; Kanniyan, 2015; Terry et al., 1995; Walter et al., 2019), mindfulness/acceptance/meditation-based interventions (Dehghani et al., 2018; Gao et al., 2024; Sánchez-Sánchez et al., 2023; Scott-Hamilton et al., 2016; Tóth et al., 2023; Yu et al., 2024; Bu et al., 2020), cognitive-behavioral/rational-emotive interventions,(Barlow and Banks, 2014; Chen et al., 2024; Tóth et al., 2023) and multicomponent/integrative interventions (Bordo et al., 2025; Terry et al., 1995; Liu, 2023; Liu et al., 2021). All included studies reported anxiety-related outcomes, although the assessment tools varied. The most frequently used instrument was the Competitive State Anxiety Inventory-2/Revised Competitive State Anxiety Inventory-2 (CSAI-2/CSAI-2R; Ali, 2015; Chen et al., 2024; Fortes et al., 2016; Davisson, 2008; Hatzigeorgiadis et al., 2009; Kanniyan, 2015; Liang et al., 2021; Sánchez-Sánchez et al., 2023; Terry et al., 1995; Tóth et al., 2023; Yu et al., 2024; Jiang, 2000; Liu, 2023; Liu et al., 2021), followed by the Sport Competition Anxiety Test (SCAT; Dehghani et al., 2018; Gao et al., 2024; Georgakaki and Karakasidou, 2017; Bu et al., 2020), Sport Anxiety Scale-2 (SAS-2; Barnicle and Burton, 2016; Scott-Hamilton et al., 2016), and State-Trait Anxiety Inventory (STAI; Barlow and Banks, 2014; Paul and Garg, 2012). Detailed study characteristics are presented in Supplementary Additional File 4.

### 3.3 Risk of bias of included studies

The methodological quality of the 24 included studies was assessed using the Cochrane Risk of Bias 2 (RoB 2) tool. The RoB 2 assessment showed that, across the five bias domains and the overall judgment, no study was rated as having a low overall risk of bias. Overall, 12 studies (50.0%) were judged as having some concerns (Barlow and Banks, 2014; Barnicle and Burton, 2016; Bordo et al., 2025; Dehghani et al., 2018; Gao et al., 2024; Georgakaki and Karakasidou, 2017; Paul and Garg, 2012; Terry et al., 1995; Yu et al., 2024; Bu et al., 2020; Jiang, 2000; Liu, 2023), whereas the remaining 12 studies (50.0%) were judged as having a high risk of bias (Ali, 2015; Chen et al., 2024; Fortes et al., 2016; Davisson, 2008; Hatzigeorgiadis et al., 2009; Kanniyan, 2015; Liang et al., 2021; Sánchez-Sánchez et al., 2023; Scott-Hamilton et al., 2016; Tóth et al., 2023; Walter et al., 2019; Liu et al., 2021).

Across individual domains, bias arising from the randomization process was most commonly rated as some concerns (83.3%), and bias due to deviations from intended interventions likewise predominantly fell into the category of some concerns (87.5%). In contrast, the risks related to missing outcome data and outcome measurement were relatively lower, with 14 studies (58.3%) rated as low risk in each of these two domains. Bias in the selection of the reported result was mainly judged as some concerns (75.0%). Overall, these findings suggest that the methodological quality of the current body of evidence was moderate to low, with the main limitations relating to the implementation of randomization procedures, control over intervention delivery, and limited transparency in outcome reporting. Detailed results of the risk-of-bias assessment are provided in the Supplementary Additional File 5.

### 3.4 Pairwise meta-analysis

**FIGURE 2:**
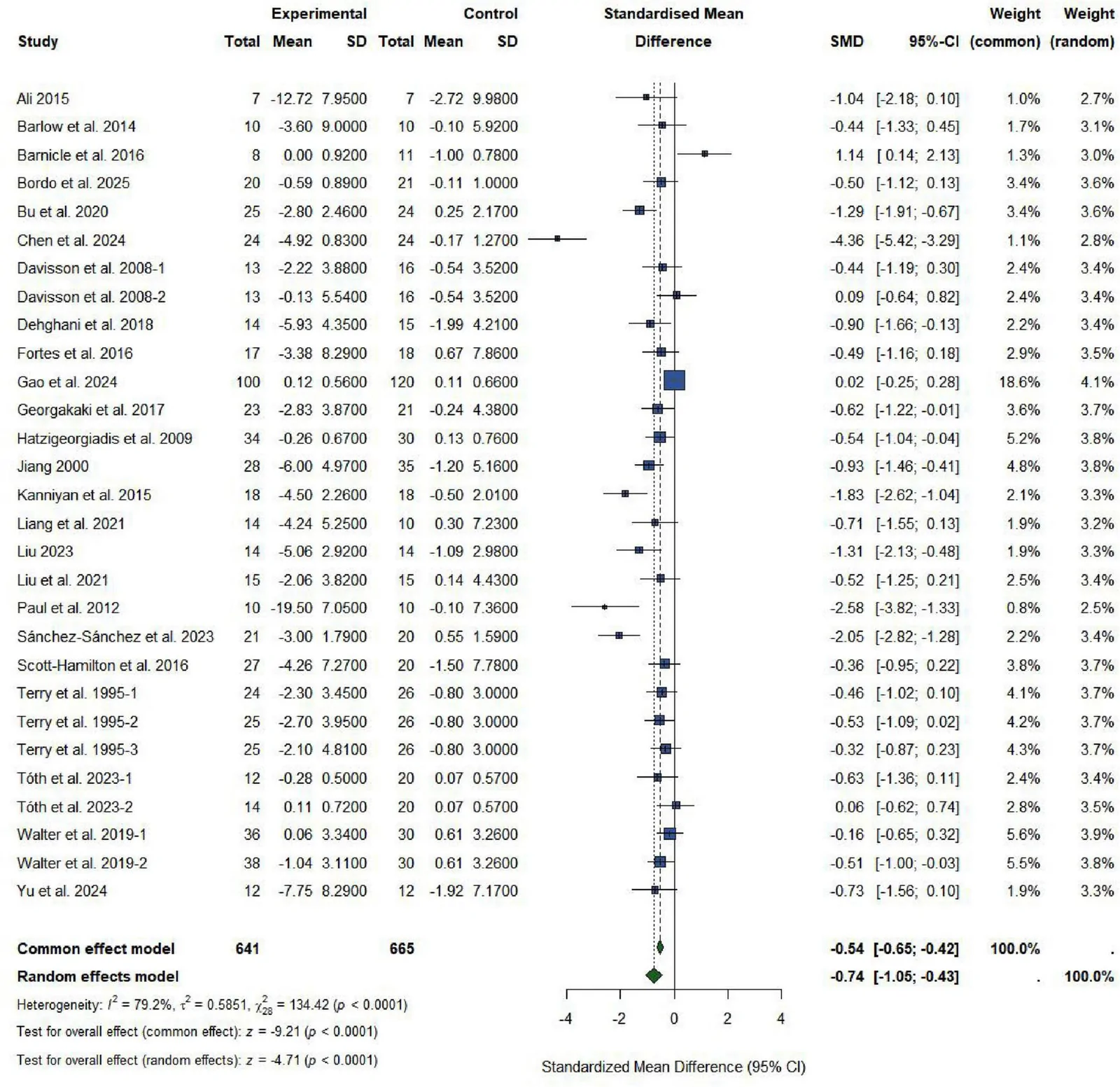
Forest plot of the pairwise meta-analysis.

As shown in Figure 2, the pairwise meta-analysis included 29 effect sizes, involving 641 participants in the intervention groups and 665 participants in the control groups. Given the presence of substantial between-study heterogeneity (I^2^ = 79.2%, *p* < 0.0001), a random-effects model was applied. The pooled results indicated that sport psychology interventions significantly reduced anxiety levels in competitive athletes compared with control conditions (SMD = −0.74, 95% CI: −1.05 to −0.43, *p* < 0.0001). Sensitivity analyses showed that, after sequential omission of each individual study, the direction and statistical significance of the pooled effect remained materially unchanged, indicating that the findings of the pairwise meta-analysis were robust. Detailed results of the sensitivity analysis are presented in the Supplementary Additional File 6.

Subgroup analyses indicated that the overall anxiety-reducing effect of sport psychology interventions was broadly consistent across delivery format and competitive level strata. Significant effects were observed for both individual and group-based interventions, with no significant between-subgroup difference (*p* = 0.973). Likewise, although the professional/provincial-level subgroup showed a relatively larger effect size, the between-subgroup difference by competitive level was not significant (*p* = 0.329). In contrast, intervention timing yielded a significant subgroup effect (*p* = 0.043), with the largest effect observed for interventions delivered during the regular training period, while immediate pre-competition interventions also showed a consistent benefit. Given the limited comparative information provided by pairwise subgroup analyses, a Bayesian network meta-analysis was further performed to compare and rank different sport psychology interventions (Supplementary Additional File 7).

Meta-regression analyses did not identify any significant linear associations between the examined covariates and effect sizes (all *p* > 0.05). These findings suggest that the observed heterogeneity may reflect unmeasured sources of variation or the limited power of meta-regression due to the relatively small number of included studies (Supplementary Additional File 8).

### 3.5 Bayesian network meta-analysis

**FIGURE 3:**
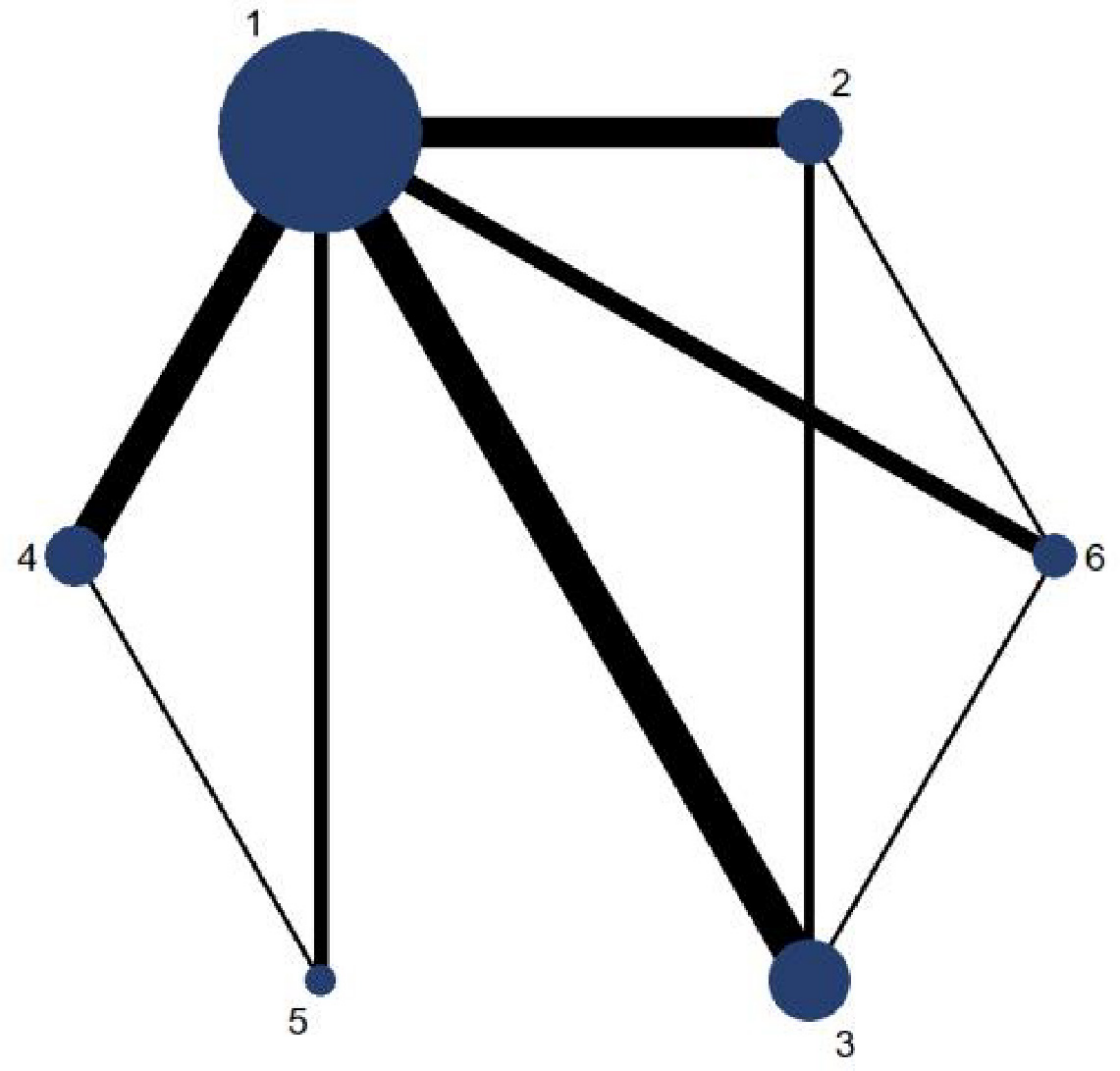
Network plot of the included interventions. Nodes 1–6 represent PC, AR, PST, MAM, CBT, and MCI, respectively. Node size is proportional to the number of participants, and edge thickness is proportional to the number of direct comparisons.

A total of six interventions (AR, CBT, MAM, MCI, PST, and PC) were included in the Bayesian network meta-analysis, forming a connected treatment network. The network plot indicated that the included interventions were linked through both direct and indirect evidence, supporting the feasibility of network meta-analysis. Variation in node size and edge thickness suggested that the amount of evidence was unevenly distributed across comparisons, with some contrasts informed mainly by direct evidence and others depending largely on indirect comparisons (Figure 3).

The league table showed that, across all pairwise comparisons, only AR, CBT, and MAM demonstrated statistically significant advantages over PC. Specifically, AR was superior to PC with an effect estimate of 0.84 (95% CrI: 0.11–1.59), CBT was superior to PC with an effect estimate of 1.73 (95% CrI: 0.68–2.81), and MAM was superior to PC with an effect estimate of 0.79 (95% CrI: 0.11–1.48). Although MCI and PST also showed trends toward improvement relative to PC, these differences did not reach statistical significance. No statistically significant differences were observed in any of the remaining pairwise comparisons between active interventions (Table 1).

**TABLE 1:** League table of the Bayesian network meta-analysis.

AR	AR	CBT	MAM	MCI	PC	PST
CBT	0.89 (−0.40, 2.19)					
MAM	−0.05 (−1.06, 0.95)	−0.94 (−2.13, 0.23)				
MCI	−0.17 (−1.26, 0.90)	−1.06 (−2.45, 0.30)	−0.12 (−1.23, 0.99)			
PC	−0.84 (−1.59, −0.11)	−1.73 (−2.81, −0.68)	−0.79 (−1.48, −0.11)	−0.67 (−1.55, 0.20)		
PST	−0.29 (−1.18, 0.59)	−1.18 (−2.43, 0.04)	−0.23 (−1.18, 0.69)	−0.12 (−1.15, 0.90)	0.56 (−0.07, 1.19)	

Positive values indicate that the row intervention was superior to the column intervention, whereas negative values indicate that the column intervention was superior to the row intervention. Bold values indicate statistically significant differences (95% CrI excluding 0).

**FIGURE 4:**
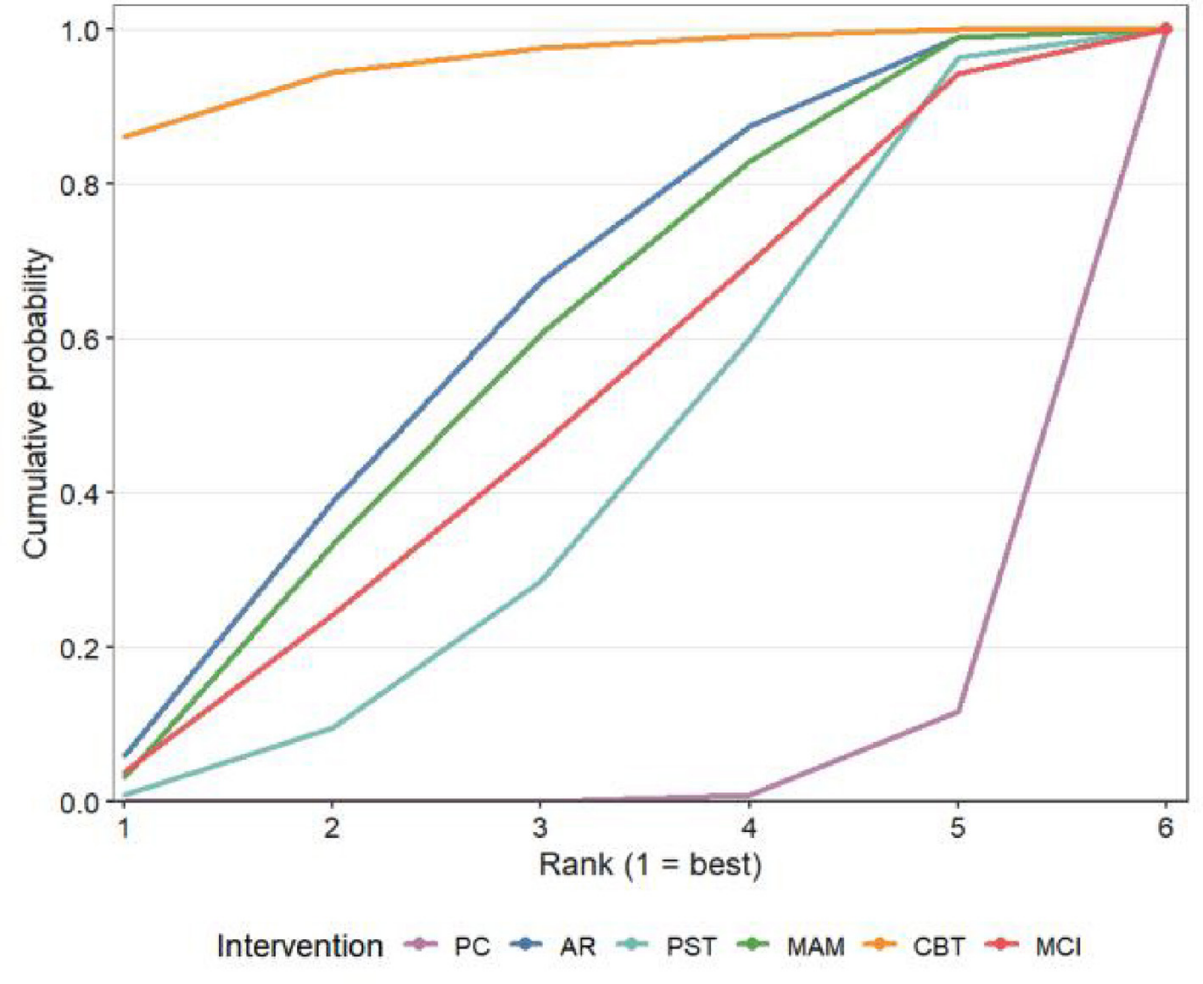
Cumulative ranking curves (SUCRA).

The ranking probability analysis showed that CBT had an 86.14% probability of being ranked first, suggesting that it was the intervention most likely to be the most effective. AR and MAM were most frequently ranked second to third, indicating relatively favorable overall efficacy. MCI and PST were mainly distributed across the fourth to fifth ranks, whereas PC had an 88.03% probability of being ranked last, suggesting that it was most likely to be the least effective intervention. Overall, the ranking results were broadly consistent with the league table, indicating that CBT, AR, and MAM were likely to have comparatively better efficacy, whereas PC ranked lowest (Figure 4).

Model diagnostics indicated adequate convergence of the Bayesian network meta-analysis, with PSRF values close to 1.00, good mixing of the trace plots, and stable posterior density distributions. Node-splitting analyses showed no significant inconsistency between direct and indirect evidence for any comparison (all *p* > 0.05), supporting the use of the consistency model (Supplementary Additional Files 9, 10).

**FIGURE 5:**
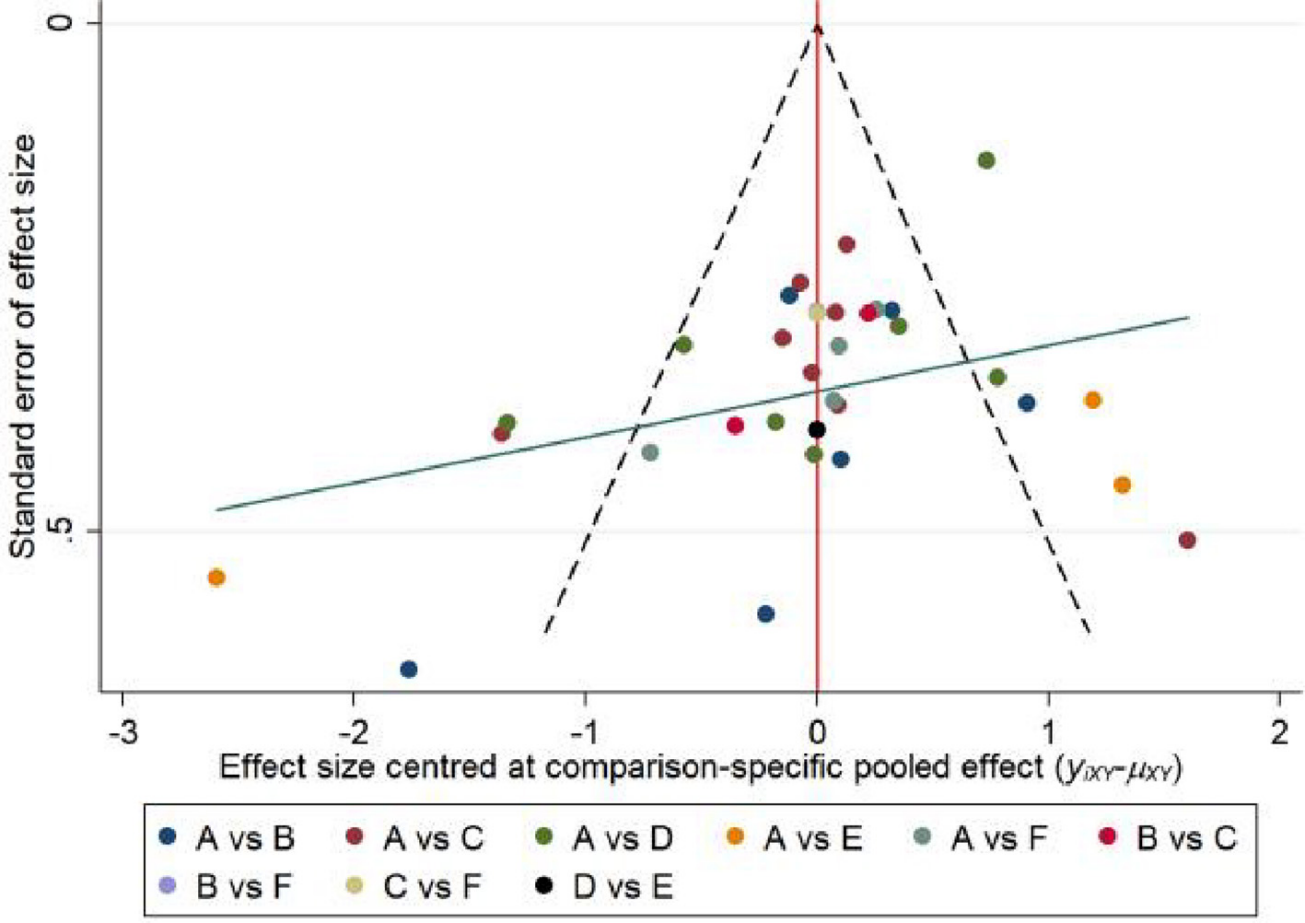
Comparison-adjusted funnel plot.

The comparison-adjusted funnel plot suggested possible small-study effects or publication bias, although the evidence was insufficient for a definitive conclusion; therefore, these findings should be interpreted cautiously in light of the network structure and overall consistency results (Figure 5).

## 4 Discussion

This study integrated evidence from pairwise meta-analysis, subgroup analysis, meta-regression, and Bayesian network meta-analysis to provide a comprehensive evaluation of the effects of different sport psychology interventions on anxiety in competitive athletes. Overall, the findings support the beneficial role of sport psychology interventions in reducing anxiety in this population. The Bayesian network meta-analysis further suggested that, relative to passive control, AR, CBT, and MAM were associated with significant benefits, and that CBT was most likely to rank as the most effective intervention under the current evidence framework. Subgroup analyses further indicated that the overall beneficial pattern was generally consistent across different delivery formats and competitive levels, whereas meta-regression did not detect significant linear relationships between the examined study-level covariates and effect sizes.

The overall finding that sport psychology interventions can reduce anxiety in competitive athletes is broadly consistent with prior pairwise meta-analytic evidence. Previous studies have generally shown that psychological interventions are beneficial for alleviating competitive anxiety in athletes. For instance, Li et al. (2025) reported in a meta-analysis that psychological interventions significantly reduced athlete anxiety. Notably, the present network meta-analysis suggested that CBT may hold a relative advantage over other intervention approaches. This finding is plausible because competitive anxiety is not merely characterized by elevated physiological arousal; it also involves maladaptive cognitive processes, including fear of failure, self-doubt, perceived evaluative threat, and disrupted attentional control. Theoretical models of competitive anxiety and anxiety–cognition interactions have consistently indicated that anxiety compromises top-down attentional control, increasing vulnerability to threat cues and task-irrelevant interference (Nieuwenhuys and Oudejans, 2012; Oudejans et al., 2011). In contrast to interventions that primarily target arousal reduction, CBT is more directly oriented toward the cognitive mechanisms underlying anxiety maintenance. By promoting cognitive restructuring, irrational belief modification, and adaptive coping training, CBT may help athletes reduce maladaptive appraisals while enhancing emotional regulation and psychological coping resources (Olmedilla et al., 2010; Lorenzo Calvo et al., 2012; Mostaan et al., 2015). Consistent with this interpretation, studies on REBT and CBT in sport psychology have emphasized that the therapeutic goal is not simply to remove stress, but to reshape how athletes interpret, appraise, and respond to stressful competitive situations (Turner, 2016; Isorna-Folgar et al., 2022; Turner and Barker, 2013).

In addition to CBT, both AR and MAM demonstrated relatively favorable effects in the present study, suggesting that interventions targeting physiological arousal regulation and those based on mindfulness and acceptance may both represent meaningful approaches to anxiety reduction in competitive athletes. The relative benefit of AR may stem primarily from its direct effect on physiological activation. Competitive anxiety is frequently accompanied by somatic manifestations such as muscle tension, elevated heart rate, and dysregulated breathing (Lundqvist and Hassmén, 2005). By attenuating excessive physiological activation, relaxation-based approaches may provide relatively rapid relief from tension and contribute to short-term emotional regulation. Consistent with this interpretation, prior studies have reported that progressive muscle relaxation can improve anxiety, stress, and heart-rate-related indicators in basketball athletes (Battaglini et al., 2022; Kudlackova et al., 2013; Parnabas et al., 2014). By contrast, the strength of MAM may lie less in suppressing anxiety itself and more in altering athletes’ relationship with anxious internal experiences. Rather than treating anxiety as something that must be completely removed, MAM-based approaches aim to enhance present-focused awareness, reduce rumination, and decrease experiential avoidance, thereby limiting the extent to which anxiety disrupts attentional control and task performance (Gardner and Moore, 2004). Previous studies have shown that MAM interventions can reduce sport-related anxiety and experiential avoidance while improving broader psychological functioning, and systematic reviews in sport psychology have generally supported the positive role of mindfulness-based interventions in both anxiety regulation and performance-related outcomes (Dehghani et al., 2018; Wang et al., 2023; Bühlmayer et al., 2017). Taken together, the differences among CBT, AR, and MAM may not be reducible to a simple hierarchy of effect sizes. Rather, they may reflect distinctions in mechanism of action and contextual suitability. AR may be particularly useful when rapid downregulation of heightened arousal is required, whereas MAM may be more relevant for developing psychological flexibility and stable attentional control through repeated training and longer-term practice (Wang et al., 2025).

A noteworthy finding from the subgroup analysis was that intervention timing may be an important modifier of treatment effects, with interventions delivered during the regular training period yielding larger effect sizes than those delivered immediately before competition. This pattern is consistent with the perspective that psychological skills are most effectively developed through systematic and sustained training rather than through isolated short-term application (Birrer and Morgan, 2010; Mujika et al., 2018). A plausible explanation is that the regular training phase offers greater opportunity for repeated practice, consolidation, and internalization of coping strategies, enabling athletes to develop and stabilize these skills under relatively low-pressure conditions before transferring them to high-pressure competition settings (Birrer and Morgan, 2010; Griffith et al., 2024). In contrast, although immediate pre-competition interventions also appeared beneficial, their effects may be concentrated more on acute state regulation (Yu et al., 2024) and may therefore be less conducive to the development of enduring anxiety-management capacities. These subgroup findings suggest that anxiety regulation should not be conceptualized solely as a pre-competition support technique, but rather as a component of the broader training support framework that can be progressively embedded within periodized preparation programs (Mujika et al., 2018; Alecu and Onea, 2025). Furthermore, meta-regression did not reveal significant linear associations between the examined covariates and effect sizes. However, this result is more appropriately interpreted as indicating that the currently available study-level variables were insufficient to account for the observed heterogeneity, rather than as evidence that intervention effects are uniformly generalizable across populations and settings.

From a practical perspective, the present findings may help inform the selection of sport psychology interventions in competitive sport settings. Under the current evidence, CBT demonstrated the highest probability of being the most effective intervention for reducing anxiety, whereas AR and MAM also showed favorable effects compared with passive control. Therefore, these interventions may be considered when designing psychological support programs for competitive athletes. CBT may be particularly appropriate when qualified sport psychologists or practitioners trained in cognitive-behavioral techniques are available, whereas AR and MAM may represent more feasible options in routine training settings because they are generally easier to implement within regular practice. In addition, the subgroup findings suggest that integrating psychological interventions into regular training periods may yield greater benefits than delivering them only immediately before competition. However, this finding should be interpreted cautiously because it was derived from subgroup analyses. Nevertheless, intervention selection should also take into account athletes’ individual characteristics, available resources, practitioner expertise, and the practical context of implementation, given the exploratory nature of the subgroup analyses and the methodological limitations of the available evidence.

Despite integrating evidence from pairwise meta-analysis, subgroup analysis, meta-regression, and Bayesian network meta-analysis, the present findings should be interpreted in light of several limitations related to both the original evidence base and the structure of the treatment network. First, substantial heterogeneity was observed in the pairwise meta-analysis, indicating meaningful variation across studies in intervention content, implementation period, participant characteristics, and anxiety assessment methods. Although standardized mean differences (SMDs) were used to improve comparability across outcome measures, the use of different anxiety assessment instruments may nevertheless have contributed to between-study heterogeneity and should be considered when interpreting the pooled estimates. Second, in the Bayesian network meta-analysis, the distribution of studies and sample sizes across intervention nodes was imbalanced, and several comparisons depended largely on indirect rather than direct evidence. Accordingly, the ranking results should be interpreted as reflecting the relative performance of interventions within the current evidence network rather than definitive conclusions. Although no statistically significant inconsistency was detected, the limited number of direct head-to-head comparisons warrants cautious interpretation of the consistency assumption. In addition, none of the included studies was judged to have an overall low risk of bias, with approximately half rated as high risk. This reduces confidence in the pooled estimates, particularly for effect sizes and comparative rankings, which may be overestimated relative to what would be expected from higher-quality trials. Accordingly, the present findings should be interpreted as preliminary and hypothesis-generating rather than as confirming the definitive superiority of any single intervention. Third, both subgroup analyses and meta-regression were exploratory and should therefore be interpreted cautiously. In particular, conclusions regarding intervention timing should be regarded as hypothesis-generating rather than confirmatory, as they were derived from subgroup analyses. Moreover, additional sources of heterogeneity, including coach support, intervention fidelity, and athletes’ baseline psychological profiles, may not have been adequately measured in the primary studies. Finally, although the included interventions differed in theoretical orientation and delivery format, they shared the common objective of reducing anxiety in competitive athletes. Nevertheless, residual differences in participant characteristics and intervention characteristics may have influenced the plausibility of the transitivity assumption. Accordingly, the comparative estimates generated by the network meta-analysis should be interpreted within the context of this underlying assumption. Future studies should therefore conduct more rigorous head-to-head randomized controlled trials, refine the classification and reporting of sport psychology interventions, and more clearly distinguish anxiety outcome domains and competitive context characteristics in order to verify the comparative advantages of different interventions and define the conditions under which they are most applicable.

## 5 Conclusion

The present study suggests that sport psychology interventions are beneficial for reducing anxiety in competitive athletes overall. The Bayesian network meta-analysis further indicates that different intervention approaches may vary in their relative effectiveness, with CBT, AR, and MAM emerging as the more promising options, and CBT appearing most likely to hold a relative advantage under the current evidence framework. Taken together, these findings support the role of psychological interventions in the management of competitive anxiety and highlight the value of integrating psychological skills training more systematically into routine training programs to support evidence-informed intervention selection in competitive sport.

## Data Availability

The original contributions presented in this study are included in this article/Supplementary material, further inquiries can be directed to the corresponding author.
