# Supplementary material for: Comparative effects of different sport psychology interventions on anxiety in competitive athletes: a Bayesian network meta-analysis
Source: Front Psychol. 2026 Aug 12;17:1842687. doi: 10.3389/fpsyg.2026.1842687 (PMC13506469; doi:10.3389/fpsyg.2026.1842687)
Supplement: Supplementary file 2 [file Table_2.DOCX]

**Comparative Effects of Different Sport Psychology Interventions on Anxiety in Competitive Athletes: A Bayesian Network Meta-analysis**

Zhongjun Chen^1^, Lian ling^1^, Songwei Zhong^1*^

^1^Faculty of Sports and Health Sciences, Guangxi Science & Technology Normal University,

Laibin, Guangxi Zhuang Autonomous Region, China.

*** Correspondence:
Songwei Zhong**
zsw864935024@163.com

**Contents**

**[Additional file 1. Search Strategy	1](#_Toc13237)**

[Table S1.1 PubMed search strategy	1](#_Toc31781)

[Table S1.2 Web of Science Core Collection search strategy	2](#_Toc29791)

[Table S1.3 Scopus search strategy	3](#_Toc770)

[Table S1.4 Embase search strategy	4](#_Toc27990)

[Table S1.5 APA PsycInfo search strategy	5](#_Toc14102)

[Table S1.7 VIP search strategy	6](#_Toc7506)

**[Additional file 2. Detailed formula-based statistical methods	7](#_Toc18199)**

**[Additional file 3. Classification of sport psychology interventions and rationale for categorization	8](#_Toc32651)**

**[Additional file 4. Characteristics of Included Studies	9](#_Toc4944)**

[Table S4.1 Study characteristics of the included studies	9](#_Toc17741)

[Table S4.2 Intervention characteristics of the included studies	12](#_Toc14692)

**[Additional file 5. Risk of bias assessment	15](#_Toc2345)**

**[Additional file 6. Leave-one-out sensitivity analysis	16](#_Toc30509)**

**[Additional file 7. Subgroup analyses of cognitive outcomes	17](#_Toc12503)**

[Figure S7.1 Type_code of anxious	17](#_Toc26022)

[Figure S7.2 Level_code of anxious	18](#_Toc6917)

[Figure S7.3 Timing_code of anxious	19](#_Toc21496)

**[Additional file 8. Meta-regression analyses	20](#_Toc24732)**

[Figure S8.1 Dose-response meta-regression for dose_min_week	20](#_Toc3111)

[Figure S8.2 Dose-response meta-regression for dose_total	20](#_Toc27982)

**[Additional file 9. Consistency and inconsistency assessments	21](#_Toc22004)**

**[Additional file 10. Local Inconsistency Test	22](#_Toc4451)**

[Figure S10.1 Trace plots and posterior density plots	23](#_Toc29157)

[Figure S10.2 Convergence diagnostics (Gelman–Rubin plots)	24](#_Toc32544)

[Figure S10.3 Heap Sort Diagram	25](#_Toc21919)

[Table S10.1 Heterogeneity assessment	25](#_Toc30784)

**Additional file 1. Search Strategy**

**Table S1.1 PubMed search strategy**

Database: PubMed

Search date: March 19, 2026

Coverage: Inception to March 19, 2026

Records retrieved: 64

| **Step** | **Query** |
| --- | --- |
| #1 | "Athletes"[Mesh] OR athlete*[tiab] OR sportsperson*[tiab] OR sportsman*[tiab] OR sportsmen[tiab] OR sportswoman*[tiab] OR player*[tiab] OR competitor*[tiab] OR "competitive athlete*"[tiab] OR "elite athlete*"[tiab] OR "professional athlete*"[tiab] OR "collegiate athlete*"[tiab] OR "student athlete*"[tiab] OR "high-performance athlete*"[tiab] OR "trained athlete*"[tiab] OR "sub-elite athlete*"[tiab] |
| #2 | "Psychology, Sports"[Mesh] OR "psychological intervention*"[tiab] OR "psychological skill*"[tiab] OR "psychological skills training"[tiab] OR PST[tiab] OR "mental skill*"[tiab] OR "mental training"[tiab] OR "mental skills training"[tiab] OR "relaxation training"[tiab] OR relaxation[tiab] OR "progressive muscle relaxation"[tiab] OR PMR[tiab] OR imagery[tiab] OR "mental imagery"[tiab] OR visualization[tiab] OR "guided imagery"[tiab] OR "self-talk"[tiab] OR "goal setting"[tiab] OR mindfulness[tiab] OR "mindfulness-based"[tiab] OR meditation[tiab] OR acceptance[tiab] OR commitment[tiab] OR MAC[tiab] OR MAIC[tiab] OR MSPE[tiab] OR "cognitive behavioral"[tiab] OR "cognitive-behavioral"[tiab] OR CBT[tiab] OR REBT[tiab] OR "rational emotive behavior*"[tiab] OR "stress inoculation"[tiab] OR "arousal regulation"[tiab] OR "anxiety management"[tiab] OR biofeedback[tiab] |
| #3 | "Anxiety"[Mesh] OR anxiet*[tiab] OR "competitive anxiety"[tiab] OR "competition anxiety"[tiab] OR "sport anxiety"[tiab] OR "state anxiety"[tiab] OR "cognitive anxiety"[tiab] OR "somatic anxiety"[tiab] OR "precompetitive anxiety"[tiab] OR "pre-competitive anxiety"[tiab] |
| #4 | randomized controlled trial[pt] OR controlled clinical trial[pt] OR random*[tiab] OR randomly[tiab] OR trial[tiab] OR RCT[tiab] |
| Final | #1 AND #2 AND #3 AND #4 |

**Table S1.2 Web of Science Core Collection search strategy**

Database: Web of Science Core Collection

Search date: March 19, 2026

Coverage: Inception to March 19, 2026

Records retrieved: 150

| **Step** | **Query** |
| --- | --- |
| #1 | TS=(athlete* OR sportsperson* OR sportsman* OR sportsmen OR sportswoman* OR player* OR competitor* OR "competitive athlete*" OR "elite athlete*" OR "professional athlete*" OR "collegiate athlete*" OR "student athlete*" OR "high-performance athlete*" OR "trained athlete*" OR "sub-elite athlete*") |
| #2 | TS=("psychological intervention*" OR "psychological skill*" OR "psychological skills training" OR PST OR "mental skill*" OR "mental training" OR "mental skills training" OR "relaxation training" OR relaxation OR "progressive muscle relaxation" OR PMR OR imagery OR "mental imagery" OR visualization OR "guided imagery" OR "self-talk" OR "goal setting" OR mindfulness OR "mindfulness-based" OR meditation OR acceptance OR commitment OR MAC OR MAIC OR MSPE OR "cognitive behavioral" OR "cognitive-behavioral" OR CBT OR REBT OR "rational emotive behavior*" OR "stress inoculation" OR "arousal regulation" OR "anxiety management" OR biofeedback) |
| #3 | TS=(anxiet* OR "competitive anxiety" OR "competition anxiety" OR "sport anxiety" OR "state anxiety" OR "cognitive anxiety" OR "somatic anxiety" OR "precompetitive anxiety" OR "pre-competitive anxiety") |
| #4 | TS=(random* OR randomly OR trial OR RCT OR "controlled trial" OR "randomized controlled trial") |
| Final | #1 AND #2 AND #3 AND #4 |

**Table S1.3 Scopus search strategy**

Database: Scopus
Search date: March 19, 2026
Coverage: Inception to March 19, 2026
Records retrieved: 154

| **Step** | **Query** |
| --- | --- |
| #1 | TITLE-ABS-KEY(athlete* OR sportsperson* OR sportsman* OR sportsmen OR sportswoman* OR player* OR competitor* OR "competitive athlete*" OR "elite athlete*" OR "professional athlete*" OR "collegiate athlete*" OR "student athlete*" OR "high-performance athlete*" OR "trained athlete*" OR "sub-elite athlete*") |
| #2 | TITLE-ABS-KEY("psychological intervention*" OR "psychological skill*" OR "psychological skills training" OR PST OR "mental skill*" OR "mental training" OR "mental skills training" OR "relaxation training" OR relaxation OR "progressive muscle relaxation" OR PMR OR imagery OR "mental imagery" OR visualization OR "guided imagery" OR "self-talk" OR "goal setting" OR mindfulness OR "mindfulness-based" OR meditation OR acceptance OR commitment OR MAC OR MAIC OR MSPE OR "cognitive behavioral" OR "cognitive-behavioral" OR CBT OR REBT OR "rational emotive behavior*" OR "stress inoculation" OR "arousal regulation" OR "anxiety management" OR biofeedback) |
| #3 | TITLE-ABS-KEY(anxiet* OR "competitive anxiety" OR "competition anxiety" OR "sport anxiety" OR "state anxiety" OR "cognitive anxiety" OR "somatic anxiety" OR "precompetitive anxiety" OR "pre-competitive anxiety") |
| #4 | TITLE-ABS-KEY(random* OR randomly OR trial OR RCT OR "controlled trial" OR "randomized controlled trial") |
| Final | #1 AND #2 AND #3 AND #4 |

**Table S1.4 Embase search strategy**

Database: Embase

Platform: Embase.com

Search date: March 19, 2026

Coverage: Inception to March 19, 2026

Records retrieved: 115

| **Step** | **Query** |
| --- | --- |
| #1 | (athlete* OR sportsperson* OR sportsman* OR sportsmen OR sportswoman* OR player* OR competitor* OR 'competitive athlete*' OR 'elite athlete*' OR 'professional athlete*' OR 'collegiate athlete*' OR 'student athlete*' OR 'high-performance athlete*' OR 'trained athlete*' OR 'sub-elite athlete*'):ti,ab,kw |
| #2 | ('psychological intervention*' OR 'psychological skill*' OR 'psychological skills training' OR pst OR 'mental skill*' OR 'mental training' OR 'mental skills training' OR 'relaxation training' OR relaxation OR 'progressive muscle relaxation' OR pmr OR imagery OR 'mental imagery' OR visualization OR 'guided imagery' OR 'self-talk' OR 'goal setting' OR mindfulness OR 'mindfulness-based' OR meditation OR acceptance OR commitment OR mac OR maic OR mspe OR 'cognitive behavioral' OR 'cognitive-behavioral' OR cbt OR rebt OR 'rational emotive behavior*' OR 'stress inoculation' OR 'arousal regulation' OR 'anxiety management' OR biofeedback):ti,ab,kw |
| #3 | (anxiet* OR 'competitive anxiety' OR 'competition anxiety' OR 'sport anxiety' OR 'state anxiety' OR 'cognitive anxiety' OR 'somatic anxiety' OR 'precompetitive anxiety' OR 'pre-competitive anxiety'):ti,ab,kw |
| #4 | (random* OR randomly OR trial OR rct):ti,ab,kw |
| Final | #1 AND #2 AND #3 AND #4 |

**Table S1.5 APA PsycInfo search strategy**

Database: APA PsycInfo

Platform: EBSCOhost

Search date: March 19, 2026

Coverage: Inception to March 19, 2026

Records retrieved: 42

| **Step** | **Query** |
| --- | --- |
| #1 | TI ( athlete* OR sportsperson* OR sportsman* OR sportsmen OR sportswoman* OR player* OR competitor* OR "competitive athlete*" OR "elite athlete*" OR "professional athlete*" OR "collegiate athlete*" OR "student athlete*" OR "high-performance athlete*" OR "trained athlete*" OR "sub-elite athlete*" ) OR AB ( athlete* OR sportsperson* OR sportsman* OR sportsmen OR sportswoman* OR player* OR competitor* OR "competitive athlete*" OR "elite athlete*" OR "professional athlete*" OR "collegiate athlete*" OR "student athlete*" OR "high-performance athlete*" OR "trained athlete*" OR "sub-elite athlete*" ) |
| #2 | TI ( "psychological intervention*" OR "psychological skill*" OR "psychological skills training" OR PST OR "mental skill*" OR "mental training" OR "mental skills training" OR "relaxation training" OR relaxation OR "progressive muscle relaxation" OR PMR OR imagery OR "mental imagery" OR visualization OR "guided imagery" OR "self-talk" OR "goal setting" OR mindfulness OR "mindfulness-based" OR meditation OR acceptance OR commitment OR MAC OR MAIC OR MSPE OR "cognitive behavioral" OR "cognitive-behavioral" OR CBT OR REBT OR "rational emotive behavior*" OR "stress inoculation" OR "arousal regulation" OR "anxiety management" OR biofeedback ) OR AB ( "psychological intervention*" OR "psychological skill*" OR "psychological skills training" OR PST OR "mental skill*" OR "mental training" OR "mental skills training" OR "relaxation training" OR relaxation OR "progressive muscle relaxation" OR PMR OR imagery OR "mental imagery" OR visualization OR "guided imagery" OR "self-talk" OR "goal setting" OR mindfulness OR "mindfulness-based" OR meditation OR acceptance OR commitment OR MAC OR MAIC OR MSPE OR "cognitive behavioral" OR "cognitive-behavioral" OR CBT OR REBT OR "rational emotive behavior*" OR "stress inoculation" OR "arousal regulation" OR "anxiety management" OR biofeedback ) |
| #3 | TI ( anxiet* OR "competitive anxiety" OR "competition anxiety" OR "sport anxiety" OR "state anxiety" OR "cognitive anxiety" OR "somatic anxiety" OR "precompetitive anxiety" OR "pre-competitive anxiety" ) OR AB ( anxiet* OR "competitive anxiety" OR "competition anxiety" OR "sport anxiety" OR "state anxiety" OR "cognitive anxiety" OR "somatic anxiety" OR "precompetitive anxiety" OR "pre-competitive anxiety" ) |
| #4 | TI ( random* OR randomly OR trial OR RCT ) OR AB ( random* OR randomly OR trial OR RCT ) |
| Final | #1 AND #2 AND #3 AND #4 |

**Table S1.6 CNKI search strategy**

Database: China National Knowledge Infrastructure

Platform: CNKI

Search date: March 19, 2026

Coverage: Inception to March 19, 2026

Records retrieved: 10

| **Step** | **Query** |
| --- | --- |
| #1 | SU=(运动员+竞技运动员+优秀运动员+精英运动员+高水平运动员+职业运动员+专业运动员+大学生运动员+学生运动员+青少年运动员+竞赛运动员) |
| #2 | SU=(心理干预+运动心理干预+心理训练+心理技能训练+心理技能+心理训练方案+心理训练项目+放松训练+渐进性肌肉放松+表象训练+心理表象+意象训练+自我对话+目标设定+正念+正念训练+正念干预+接纳承诺+接纳与承诺疗法+MAC+MAIC+MSPE+认知行为干预+认知行为疗法+CBT+理性情绪疗法+REBT+唤醒调节+焦虑管理+生物反馈) |
| #3 | SU=(焦虑+竞赛焦虑+竞技焦虑+比赛焦虑+状态焦虑+认知焦虑+躯体焦虑+赛前焦虑+临赛焦虑) |
| #4 | SU=(随机对照试验+随机对照+随机分组+随机分配+对照试验+对照研究+随机试验) |
| Final | #1 AND #2 AND #3 AND #4 |

**Table S1.7 VIP search strategy**

Database: Chinese Science and Technology Journal Database

Platform: VIP

Search date: March 19, 2026

Coverage: Inception to March 19, 2026

Records retrieved: 45

| **Step** | **Query** |
| --- | --- |
| #1 | K=("运动员" + "竞技运动员" + "优秀运动员" + "精英运动员" + "高水平运动员" + "职业运动员" + "专业运动员" + "大学生运动员" + "学生运动员" + "青少年运动员" + "竞赛运动员") |
| #2 | K=("心理干预" + "运动心理干预" + "心理训练" + "心理技能训练" + "心理技能" + "心理训练方案" + "心理训练项目" + "放松训练" + "渐进性肌肉放松" + "表象训练" + "心理表象" + "意象训练" + "自我对话" + "目标设定" + "正念" + "正念训练" + "正念干预" + "接纳承诺" + "接纳与承诺疗法" + "MAC" + "MAIC" + "MSPE" + "认知行为干预" + "认知行为疗法" + "CBT" + "理性情绪疗法" + "REBT" + "唤醒调节" + "焦虑管理" + "生物反馈") |
| #3 | K=("焦虑" + "竞赛焦虑" + "竞技焦虑" + "比赛焦虑" + "状态焦虑" + "认知焦虑" + "躯体焦虑" + "赛前焦虑" + "临赛焦虑") |
| #4 | （未报告，建议补充随机对照试验检索模块） |
| Final | #1 AND #2 AND #3 |

**Additional file 2. Detailed formula-based statistical methods**

1. Data Collection
For studies that reported only the mean and standard deviation before and after the intervention but did not report the standard deviation of the difference, this study calculated the difference data using the following formula:
1) Mean:

$$\text{Mea}\text{n}_{\text{change}}\text{=Mea}\text{n}_{\text{post}}\text{−Mea}\text{n}_{\text{pre}}$$

2) Standard deviation:

$$\text{S}\text{D}_{\text{change}}\text{=}\sqrt{\text{S}\text{D}_{\text{pre}}^{\text{2}}\text{+S}\text{D}_{\text{post}}^{\text{2}}\text{−2×R×S}\text{D}_{\text{pre}}\text{×S}\text{D}_{\text{post}}}$$

2. Data Transformation
For trials comprising multiple intervention arms that share the same control group, this study included them as a single multi-arm trial in the network meta-analysis, rather than splitting them into multiple independent two-arm comparisons. This approach preserves the relevant structure of within-study comparisons and avoids overestimation of precision resulting from the repeated use of the shared control group. Suppose there are two experimental arms, A and B, within the same study that need to be combined; both belong to the same network node. Their sample sizes, means, and standard deviations are: n_1_, x_1_, SD_1_; n_2_, x_2_, SD_2_.
1) Sample size:

$$\text{n}_{\text{comb}}\text{=}\text{n}_{\text{1}}\text{+}\text{n}_{\text{2}}$$

2) Mean:

$${\bar{\text{x}}}_{\text{comb}}\text{=}\frac{\text{n}_{\text{1}}{\bar{\text{x}}}_{\text{1}}\text{+}\text{n}_{\text{2}}{\bar{\text{x}}}_{\text{2}}}{\text{n}_{\text{1}}\text{+}\text{n}_{\text{2}}}$$

3) Standard deviation:

$$\text{S}\text{D}_{\text{comb}}\text{=}\sqrt{\frac{\left( \text{n}_{\text{1}}\text{−1} \right)\text{S}\text{D}_{\text{1}}^{\text{2}}\text{+}\left( \text{n}_{\text{2}}\text{−1} \right)\text{S}\text{D}_{\text{2}}^{\text{2}}\text{+}\frac{\text{n}_{\text{1}}\text{n}_{\text{2}}}{\text{n}_{\text{1}}\text{+}\text{n}_{\text{2}}}\left( {\bar{\text{x}}}_{\text{1}}\text{−}{\bar{\text{x}}}_{\text{2}} \right)^{\text{2}}}{\text{n}_{\text{1}}\text{+}\text{n}_{\text{2}}\text{−1}}}$$

For multi-arm trials, first convert the effects of each intervention group within the same study into comparisons relative to a common reference arm, and enter the data in the long-form relative effect format required by GEMTC. Specifically, for each three-arm study, retain two treatment comparisons relative to the common reference arm and add one row of reference arm data; the effect size for the reference arm is recorded as missing, and its standard error is estimated by taking the square root of the covariance between the two comparison effects, using the following formula:

$$\text{S}\text{E}_{\text{base}}\text{=}\sqrt{\text{S}\text{E}_{\text{1}}\text{×S}\text{E}_{\text{2}}\text{×ρ}}$$

In accordance with relevant methodological recommendations, this study sets the correlation coefficient ρ to 0.5.

**Additional file 3. Classification of sport psychology interventions and rationale for categorization**

| **Study** | **Main intervention components** | **Primary theoretical orientation** | **Final classification** | **Rationale for classification** |
| --- | --- | --- | --- | --- |
| Ali 2015, Liang et al., 2020, Paul and Garg, 2012, Terry et al., 1995, 姜镇英, 2000 | Muscle relaxation, breathing regulation, arousal reduction | Physiological arousal regulation | AR | because the core aim was to reduce somatic tension and physiological arousal. |
| Barnicle and Burton, 2016, Fortes, 2016, H., 2008, Hatzigeorgiadis et al., 2009, Kanniyan, 2015, Terry et al., 1995, Walter et al., 2019 | Cognitive restructuring, irrational belief modification, coping training | Cognitive-behavioral theory | CBT | because cognitive appraisal and restructuring were the dominant therapeutic components. |
| Dehghani et al., 2018, Gao et al., 2024, Sánchez-Sánchez et al., 2023, Scott-Hamilton et al., 2016, Tóth et al., 2023, Yu et al., 2024, 卜丹冉 et al., 2020 | Mindfulness practice, acceptance, present-moment awareness, experiential acceptance | Mindfulness- and acceptance-based approach | MAM | because mindfulness, acceptance, or attentional regulation formed the primary framework. |
| Barlow and Banks, 2014, Chen et al., 2024, Tóth et al., 2023 | Goal setting, imagery, self-talk, concentration, confidence training | Psychological skills training | PST | because the intervention mainly targeted mental skills used in sport performance contexts. |
| Bordo et al., 2025, Terry et al., 1995, 刘华茹, 2023, 刘书强 et al., 2021 | Combination of relaxation, imagery, self-talk, goal setting, cognitive strategies | Mixed theoretical framework | MCI | because the intervention combined multiple psychological techniques without one clearly dominant theoretical orientation. |

***Abbreviations:***AR = arousal regulation/relaxation-based intervention; CBT = cognitive-behavioral intervention; MAM = mindfulness- and acceptance-based intervention; PST = psychological skills training; MCI = multicomponent intervention.

**Additional file 4. Characteristics of Included Studies**

**Table S4.1 Study characteristics of the included studies**

| **Study** | **Country** | **Design** | **Participants/Sport level** | **Sport** | **Timing** | **Sample size** | **Age (years)** | **Sex**  **(M/F)** | **Outcome**  **(s)** |
| --- | --- | --- | --- | --- | --- | --- | --- | --- | --- |
| Ali 2015 | Algeria | RCT | Elite male football players from ASO Chlef club | Football | Training/pre-competition | 14 | NR | 14/0 | CSAI-2 |
| Barlow et al. 2014 | UK | RCT | High-level female netball players (professional/regional amateur) | Netball | Brief pre-competition | 20 | 18–30 | 0/20 | STAI-State |
| Barnicle et al. 2016 | USA | RCT | NCAA Division I female soccer players | Soccer | In-season | 19 | NR | 0/19 | SAS-2 |
| Bordo et al. 2025 | Italy | RCT | Competitive tennis players recruited from clubs | Tennis | Training/season period | 45 randomized; 41 analyzed | IG: 25.60±14.34; CG: 24.48±11.71 | 23/18 | IPPS-48 |
| Bu et al. 2020 | China | RCT | Elite provincial badminton athletes | Badminton | Training period | 49 | IG: 19.40±2.33; CG: 19.63±2.26 | 29/20 | SCAT |
| Chen et al. 2024 | China | RCT | Competitive Latin dance athletes with high anxiety | Latin dance | Training + simulated competition | 48 | 23–25 | 24/24 | CSAI-2 |
| Davisson et al. 2008 | USA | RCT (4-arm) | NCAA Division III swimmers | Swimming | In-season | 60 randomized; 56 completed | 18–22 | 27/33 | CSAI-2 |
| Dehghani et al. 2018 | Iran | RCT | Female university basketball athletes | Basketball | Preparation/training period | 31 | IG: 23.44±0.49; CG: 22.34±0.34 | 0/31 | SCAT |
| Fortes et al. 2016 | Brazil | RCT | State-level adolescent male swimmers | Swimming | Training/pre-competition | 35 analyzed | 15.93±0.98 | 35/0 | CSAI-2R |
| Gao et al. 2024 | China | RCT | College athletes from multiple sports | Multiple sports | Training/season period | 288 randomized; 220 completed | 19.4±1.5 | 181/107 | STAI / SCAT |
| Georgakaki et al. 2017 | Greece | RCT | Competitive swimmers from a club | Swimming | Training period | 44 | 19.8±2.16 | 23/21 | SCAT |
| Hatzigeorgiadis et al. 2009 | Greece | RCT | Adolescent competitive tennis players with regional/national experience | Tennis | Brief pre-competition | 72 randomized; 64 analyzed | 13.47±1.78 | 36/36 | CSAI-2R |
| Jiang 2000 | USA | RCT | High school swimmers | Swimming | Post-training recovery period | 70 enrolled; 58 completed | 14–17 | 42/28 | CSAI-2 |
| Kanniyan et al. 2015 | Saudi Arabia | RCT | Junior football players participating in inter-school competitions | Football | Training period | 36 | IG: 16.4±1.8; CG: 17.1±1.40 | NR | CSAI-2 |
| Liang et al. 2021 | China | RCT | University track-and-field athletes preparing for competition | Track and field | Training/pre-competition | 24 | IG: 20.85±1.46; CG: 21.00±1.16 | 12/11 | CSAI-2 |
| Liu 2023 | China | RCT | University basketball players in regular training | Basketball | Training period | 28 | 20.57±1.61 | 16/12 | CSAI-2 |
| Liu et al. 2021 | China | RCT | High-level athletes from the Chinese national sailing/windsurfing team | Sailing/Windsurfing | Pre-competition preparation | 30 | 19.8±2.6 | 12/18 | CSAI-2 |
| Paul et al. 2012 | India | RCT (3-arm) | University/state/national-level basketball players with elevated anxiety | Basketball | Short-term training period | 30 | 21.13±2.82 | 17/13 | STAI |
| Sánchez-Sánchez et al. 2023 | Spain | RCT | Federated university athletes from multiple sports | Multiple sports | Training period | 41 | 22.83±5.62 | 27/14 | CSAI-2R |
| Scott-Hamilton et al. 2016 | Australia | RCT | Competitive club cyclists | Cycling | Training period | 60 randomized; 47 analyzed | IG: 38.96±12.40; CG: 40.65±10.88 | 42/5 | SAS-2 |
| Terry et al. 1995 | UK | RCT (4-arm) | Nationally ranked junior tennis players | Tennis | Immediately before competition | 100 | 13.9±1.8 | 42/58 | CSAI-2 |
| Tóth et al. 2023 | Hungary | Parallel controlled trial | Youth ice hockey players from the same club | Ice hockey | Training period | 46 | 18.04±1.83 | 36/10 | CSAI-2 |
| Walter et al. 2019 | Germany | RCT (3-arm) | Sub-elite/youth elite athletes from multiple sports | Multiple sports | Training period | 117 randomized; 104 entered intervention | 16.0±1.8 | 62/55 | WAI-S |
| Yu et al. 2024 | China | RCT | Short-distance runners with elevated pre-competition anxiety | Sprinting | Pre-competition period | 24 | 22.46±1.35 | 14/10 | CSAI-2 |

***Abbreviations:*** *Competitive State Anxiety Inventory-2 (CSAI-2); Revised Competitive State Anxiety Inventory-2 (CSAI-2R); State-Trait Anxiety Inventory (STAI); State subscale of the State-Trait Anxiety Inventory (STAI-State); Sport Anxiety Scale-2 (SAS-2); Inventaire de Performance Psychologique et Sportive-48 (IPPS-48); Sport Competition Anxiety Test (SCAT); Wettkampf-Angstinventar–State (WAI-S).*

**Table S4.2 Intervention characteristics of the included studies**

| **Study** | **Intervention category** | **Intervention description** | **Provider/Delivery mode** | **Frequency** | **Session duration** | **Total duration** | **Comparator** | **Control type** |
| --- | --- | --- | --- | --- | --- | --- | --- | --- |
| Ali 2015 | Relaxation | Progressive muscle relaxation (Jacobson) | Researcher-guided/group-based | NR | 10 min | 20 sessions over 6 weeks | Usual training without relaxation | Passive usual-care control |
| Barlow et al. 2014 | Coaching/Solution-focused intervention | Solution-focused coaching based on emotional intelligence profile | Coach/researcher-guided | Single session | 30 min | One-off intervention | No intervention | Blank/no-intervention control |
| Barnicle et al. 2016 | Mental skills training | Individualized mental skills training (goal setting, self-talk, relaxation, etc.) | Individualized guided training | ~1 session/week | 45 min | 12 weeks | Usual training without formal MST | Passive usual-care control |
| Bordo et al. 2025 | App-based multicomponent intervention | App-based breathing, relaxation, imagery, and mindfulness-based body awareness plus psychologist support | App + online psychologist support | Daily app practice | 3–7 min | App practice for 8 weeks + online meeting every 2 weeks (20 min) | No app use | Wait-list control |
| Bu et al. 2020 | Mindfulness | MAIC mindfulness training | Instructor-guided | 1 session/week | 90 min | 7 weeks | No intervention | Wait-list control |
| Chen et al. 2024 | Systematic desensitization | Muscle relaxation, imaginal desensitization, in vivo/simulated exposure, suggestion-based relaxation | Therapist-guided | 2 sessions/week | 20–30 min | 8 weeks | Routine training only | Passive usual-care control |
| Davisson et al. 2008 | Imagery/Goal setting/Relaxation | Imagery, goal setting, or PMR relaxation | Instructor-guided | 2 sessions/week | 5–10 min | 5 weeks | No training | Blank/no-intervention control |
| Dehghani et al. 2018 | Mindfulness-based program | Mindfulness-Acceptance-Commitment (MAC) program | Instructor-guided | ~1 session/week | 90 min | 8 weeks | No intervention | Wait-list control |
| Fortes et al. 2016 | Imagery | Cognitive-general mental training using video-based imagery | Structured guided training | 3 sessions/week | 10 min | 8 weeks | Advertisement videos | Attention control |
| Gao et al. 2024 | Mobile mindfulness | Therapist-guided mobile mindfulness intervention based on MBSR | Mobile app + therapist support | Daily | ~15 min | 43 days | Health education materials | Attention control |
| Georgakaki et al. 2017 | Self-talk | Motivational self-talk training | Instructor-guided | 1 session/week | 90 min | 5 weeks | No training | Blank/no-intervention control |
| Hatzigeorgiadis et al. 2009 | Self-talk | Motivational self-talk training | Structured guided training | NR | NR | 5 sessions total, including 3 training sessions | Brief tactical instruction without self-talk | Attention control |
| Jiang 2000 | Relaxation/Breathing meditation | Breathing meditation/relaxation training | Guided recovery-based practice | 5 sessions/week | 25 min | 4 weeks | Quiet rest after training | Non-specific psychological activity control |
| Kanniyan et al. 2015 | Self-talk | Positive self-talk training | Guided practice | 3–5 days/week | 10–15 min | 8 weeks | Routine training only | Passive usual-care control |
| Liang et al. 2021 | Relaxation | Progressive relaxation training | Guided practice | 2 sessions/week | 30 min | 4 weeks | Usual post-training activity | Passive usual-care control |
| Liu 2023 | Multicomponent psychological intervention | Group counseling with psychological skills training, music therapy, and tailored exercise plans | Group-based counseling | NR | NR | NR | Routine psychological counseling | Non-specific psychological activity control |
| Liu et al. 2021 | Psychoeducation + biofeedback | Multi-component psychological intervention: psychoeducation plus biofeedback-based skills training | Instructor-guided/biofeedback-assisted | Psychoeducation once/week + biofeedback twice/week | 45–60 min | 4 weeks | Wait-list control | Wait-list control |
| Paul et al. 2012 | Biofeedback | Heart rate variability biofeedback | Guided biofeedback training | Daily | 20 min | 10 consecutive days | No-treatment control | Blank/no-intervention control |
| Sánchez-Sánchez et al. 2023 | Mindfulness/meditation | Flow Meditation mindfulness intervention | Instructor-guided + home practice | 1 session/week | 90 min | 10 weeks + daily home practice | Wait-list control | Wait-list control |
| Scott-Hamilton et al. 2016 | Mindfulness | Cyclist-specific mindfulness training based on MiCBT | Group session + home practice | Weekly | NR | 8 weeks + daily home practice | Wait-list control | Wait-list control |
| Terry et al. 1995 | Mental skills | Mental rehearsal, centering, or combined intervention | Brief guided session | Single session | NR | Immediately before competition | Concentration grid task | Attention control |
| Tóth et al. 2023 | REBT/Mindfulness | REBT or mindfulness-based group intervention | Group-based intervention | 1 session/week | 60 min | 8 weeks | Wait-list control | Wait-list control |
| Walter et al. 2019 | Self-talk | Self-talk training (short-term or long-term format) | Structured guided training | STI: 3 sessions/week; LTI: 3 sessions/week | STI: 60 min; LTI: 20 min | STI: 1 week; LTI: 8 weeks | No self-talk training | Wait-list control |
| Yu et al. 2024 | Mindfulness | MAIC mindfulness training with daily breathing practice | Instructor-guided + home practice | 1 session/week + daily practice | 60 min + 15 min breathing/day | 7 weeks | Quiet sitting/free mind-wandering | Non-specific psychological activity control |

**Additional file 5. Risk of bias assessment**

**Additional file 6. Leave-one-out sensitivity analysis**

**Additional file 7. Subgroup analyses of cognitive outcomes**

**Figure S7.1 Type_code of anxious**

**Figure S7.2 Level_code of anxious**

**Figure S7.3 Timing_code of anxious**

**Additional file 8.** **Meta-regression analyses**

**Figure S8.1 Dose-response meta-regression for dose_min_week**

**Figure S8.2 Dose-response meta-regression for dose_total**

**Additional file 9. Consistency and inconsistency assessments**

| Outcoms | Model | DIC | pD | Residual Deviance | SD (95%CrI) | I^2^ |
| --- | --- | --- | --- | --- | --- | --- |
| Anxiety | RE Consistency model | 55.14 | 24.45 | 30.69 | 0.84(95%CrI 0.55-1.24) | 12% |
|  | RE UME Model | 55.14 | 24.46 | 30.68 | 0.84(95%CrI 0.55-1.24) | 12% |

The random-effects consistency model and the random-effects UME model showed nearly identical fit statistics (DIC: 55.14 vs 55.14; pD: 24.45 vs 24.46; residual deviance: 30.69 vs 30.68), with the same heterogeneity estimate and I² value [SD = 0.84 (95% CrI 0.55–1.24), I² = 12%]. These findings suggest that allowing for inconsistency did not improve model fit, indicating no evidence of substantial global inconsistency in the network. Therefore, the consistency model was considered appropriate for the primary analysis.

**Additional file 10. Local Inconsistency Test**

Local inconsistency was assessed using the node-splitting approach. No statistically significant inconsistency was detected in any of the split comparisons, as all P values were greater than 0.05 (MCI vs AR: P = 0.994; PST vs AR: P = 0.284; MAM vs CBT: P = 0.482; PST vs MCI: P = 0.794). In addition, the direct and indirect estimates were generally comparable and their 95% credible intervals substantially overlapped, supporting the assumption of local consistency within the network.

***Abbreviations:*** *AR, arousal regulation interventions; CBT, cognitive-behavioral therapy; MAM, mindfulness/acceptance/meditation-based interventions; MCI, multicomponent interventions; PST, psychological skills training; PC, passive control.*

**Figure S10.1 Trace plots and posterior density plots**

***Abbreviations:*** *AR, arousal regulation interventions; CBT, cognitive-behavioral therapy; MAM, mindfulness/acceptance/meditation-based interventions; MCI, multicomponent interventions; PST, psychological skills training; PC, passive control.*

**Figure S10.2 Convergence diagnostics (Gelman–Rubin plots)**

***Abbreviations:*** *AR, arousal regulation interventions; CBT, cognitive-behavioral therapy; MAM, mindfulness/acceptance/meditation-based interventions; MCI, multicomponent interventions; PST, psychological skills training; PC, passive control.*

**Figure S10.3 Heap Sort Diagram**

***Abbreviations:*** *AR, arousal regulation interventions; CBT, cognitive-behavioral therapy; MAM, mindfulness/acceptance/meditation-based interventions; MCI, multicomponent interventions; PST, psychological skills training; PC, passive control.*

**Table S10.1 Heterogeneity assessment**

| **Comparison** | **No. direct studies (k)** | **I²**  **(pairwise, %)** | **I²**  **(network, %)** | **Pooled pairwise effect** | **Indirect**  **(back-calculated)** | **Pooled network effect** |
| --- | --- | --- | --- | --- | --- | --- |
| MCI vs AR | 1 | 0.0 | 0.0 | 0.16 (-1.7, 2.0) | 0.18 (-1.2, 1.5) | 0.17 (-0.90, 1.3) |
| PC vs AR | 6 | 61.4 | 60.9 | 0.88 (0.11, 1.7) | NA | 0.84 (0.11, 1.6) |
| PST vs AR | 2 | 0.0 | 39.8 | -0.18 (-1.5, 1.1) | 0.66 (-0.53, 1.9) | 0.29 (-0.59, 1.2) |
| MAM vs CBT | 1 | NR | 0.0 | 0.63 (-1.2, 2.5) | 1.2 (-0.38, 2.7) | 0.94 (-0.24, 2.1) |
| PC vs CBT | 3 | 94.7 | 94.7 | 1.7 (0.59, 2.8) | NA | 1.7 (0.68, 2.8) |
| PC vs MAM | 7 | 88.2 | 89.1 | 0.73 (0.042, 1.4) | NA | 0.79 (0.11, 1.5) |
| PC vs MCI | 4 | 12.8 | 13.7 | 0.65 (-0.26, 1.6) | NA | 0.67 (-0.21, 1.6) |
| PST vs MCI | 1 | NR | 0.0 | -0.054 (-1.9, 1.8) | 0.19 (-1.0, 1.4) | 0.12 (-0.91, 1.1) |
| PST vs PC | 8 | 68.5 | 68.9 | -0.45 (-1.1, 0.20) | NA | -0.56 (-1.2, 0.080) |

***Abbreviations:*** *AR, arousal regulation interventions; CBT, cognitive-behavioral therapy; MAM, mindfulness/acceptance/meditation-based interventions; MCI, multicomponent interventions; PST, psychological skills training; PC, passive control.*
